# Cancer Incidence Trend in the Hebei Spirit Oil Spill Area, from 1999 to 2014: An Ecological Study

**DOI:** 10.3390/ijerph15051006

**Published:** 2018-05-17

**Authors:** Kyung-Hwa Choi, Myung-Sook Park, Mina Ha, Jong-Il Hur, Hae-Kwan Cheong

**Affiliations:** 1 Taean Environmental Health Center, Taean, Chungnam 32148, Korea; rosach72@hanmail.net (K.-H.C.); pms1816@gmail.com (M.-S.P.); gsjongil@korea.kr (J.-I.H.); 2 Department of Preventive Medicine, Dankook University College of Medicine, Cheonan, Chungnam 31116, Korea; minaha@dku.edu; 3 Department of Social and Preventive Medicine, Sungkyunkwan University School of Medicine, Suwon, Gyeonggi 16419, Korea

**Keywords:** Hebei Spirit oil spill, cancer incidence, national cancer registry, long-term health effects

## Abstract

The Hebei Spirit oil spill (HSOS) occurred in the Republic of Korea on 7 December 2007. We aimed to describe the cancer incidence trend in Taean County before and after the oil spill. Five major cancers and leukemia were analyzed. Cancer incidence data were obtained from the Korean National Cancer Center. We compared the standardized incidence rates in Taean with those observed nationwide and selected three coastal areas. Joinpoint regression analysis was used to examine the trends in the average annual percent change and perform comparisons. The incidence rate of prostate cancer increased from 2007 to 2009 at an annual average of 39.3% (95% confidence interval (CI): −25.9, 161.8), 13.5% (95% CI: 11.7, 15.4), and 15.6% (95% CI: 11.9, 19.5), respectively, in Taean, nationwide, and in the coastal areas. The incidence of leukemia among women increased at an annual average of 9.5% (95% CI: −26.6, 63.4) in Taean and 0.6% (95% CI: 0.2, 0.9) nationwide; the rate decreased by 1.9% (95% CI: −12.8, 10.4) in the coastal areas. The trends between Taean County and the coastal areas differed only for prostate cancer (*p* = 0.0004). The incidence of prostate cancer among Taean County residents has increased since the HSOS.

## 1. Introduction

The Hebei Spirit oil spill (HSOS) occurred in the western coastal area of the Korean peninsula on 7 December 2007 [1], and was the worst of its kind in Korean waters [1]. In Taean, an oil band covered the coast that is very close to a residential area for one month [2]. Taean residents, in addition to over one million volunteers, and military and marine officers performed clean-up work for seven or eight months [2,3]. However, in the first few days after the oil spill, clean-up workers were not appropriately protected [3].

The spilled oil contained volatile organic compounds (VOCs), polycyclic aromatic hydrocarbons (PAHs), and heavy metals [1]. Crude oil VOCs include benzene, toluene, ethylbenzene, and xylene (BTEX), which are group 1 or 2 human carcinogens according to the International Agency for Research on Cancer (IARC) [4]. PAHs are easily absorbed by the respiratory tract and cause damage to the red blood cells [1]. The IARC has classified some PAHs, such as bezo[*a*]pyrene, as group 1 human carcinogens, and benz[*a*]anthracene, dibenz[*a,h*]anthracene, chrysene, naphthalene, bezo[*b*]fluoranthene, bezo[*k*]fluoranthene, and indeno[1,2,3-*cd*]pyrene as group 2 human carcinogens [4,5].

The HSOS continues to affect Taean County residents and clean-up workers, with the effects manifesting as various adverse health events across Korea, including acute-stage physical [3,5,6,7,8,9] and mental health problems [2,10,11,12] as well as mid- and long-term health problems [13,14]. In a study conducted 1.5 years [13] after the spill, the oxidative DNA stress marker levels, which could be indicative of cancer risk, were higher in the high-exposure group than in the low-exposure group; the effect persisted for 6 years after the spill [14]. Chromosome damage was observed among fishermen in studies conducted two and six years after the Prestige oil spill in Spain [15,16,17], and cancer risk modeling was performed after the British Petroleum Deepwater Horizon (BPDH) oil spill in the United States of America (USA) [18].

While oil spills have occurred across the world, studies on the associated mid- and long-term health effects have been reported only in three countries: Spain (Prestige oil spill), the USA (Exxon Valdez oil spill [19,20,21] and the BPDH oil spill [18,22,23,24,25,26,27,28,29,30,31,32,33]), and Korea (HSOS) [2,3,5,6,7,8,9,10,11,12,13,14]. There have been concerns pertaining to the potential development of cancers in the affected areas; however, few studies have focused on the cancer incidence among residents after the oil spill despite their consequent exposure to carcinogenic substances. These findings suggest the need for longitudinal studies on the cancer incidence in the affected areas. In this study, the authors aimed to assess the cancer incidence rate in the area affected by the HSOS and to compare it with that observed in other areas, before and after the oil spill.

## 2. Materials and Methods

### 2.1. Study Areas

#### 2.1.1. Exposure Area

**Figure 1:**
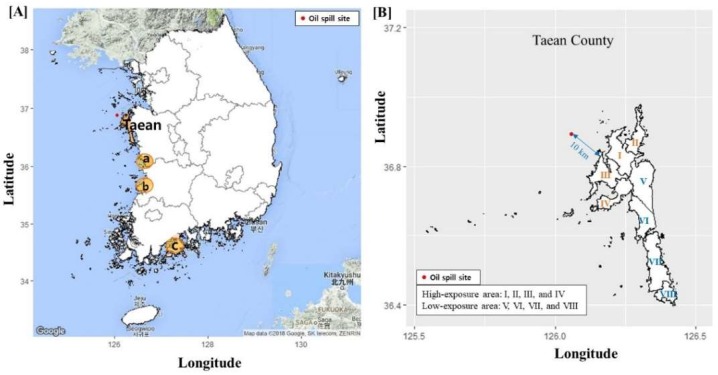
Map of Taean County and comparison of the coastal areas (a, b, and c) in Korea. (**A**) Comparison areas, “a”: Same province and same coastal area, “b”: Neighboring province and same coastal area, and “c”: Different province and different coast. The areas for comparison were selected based on their similarities to Taean County in terms of their characteristics and population size. (**B**) Taean County according to exposure level. I: Sowon, II: Wonbuk, III: Iwon, IV: Geunheung, V: Taean-eup, VI: Nam, VII: Anmyeon, and VIII: Gonam. The maps were drawn using Google maps (2018) [35] and ggplot2 in R package [36].

Taean County—the site of the HSOS—has eight towns, and we classified them into two areas of exposure (Figure 1). Four towns (Sowon, Wonbuk, Geunheung, and Iwon) which face the oil-contaminated coast and are located at a distance of less than 15 km from the contaminated shore line were classified as high-exposure areas. The low-exposure areas comprised the remaining four towns (Taean-eup, Anmyeon, Gonam, and Nam), which include both inland (Taean-eup) and coastal areas; the distance to the contaminated shore line is 10 to 45 km. Further details have been described in previous studies [2,34]. The mid-year population sizes (proportion of elderly adults) in 2007 were 9826 (21.3%) and 21,844 (13.2%) among men, and 9691 (30.4%) and 21,681 (19.7%) among women, in the high-exposure and low-exposure areas, respectively.

#### 2.1.2. Comparison Areas

The observed cancer incidence rate and trend were compared with those observed nationwide and in similar coastal areas. We selected three coastal areas which had a population size that was similar to that of Taean County, with similar regional characteristics [37]. Area “a” is located in the same province and the same coastal area, area “b” in the neighboring province and the same coastal area, and area “c” in a different province and on a different coast (Figure 1). The mid-year population sizes (proportion of elderly) in 2007 were 49,130,354 (10.2%), 63,398 (19.7%), 61,899 (25.1%), 62,561 (23.7%), and 79,829 (30.8%) in Korea, Taean County, area “a”, area “b”, and area “c”, respectively.

### 2.2. Age-Standardized Cancer Incidence

Five major cancers and leukemia (C91−C95) were selected for analysis. The five major cancers for Korean men are those of the stomach (C16), lung (C33−C34), colon (C18−C20), liver (C22), and prostate (C61); and those for Korean women are thyroid (C73), breast (C50), colon (C18−C20), stomach (C16), and lung (C33−C34) cancers [38]. Data on the cancer cases in each area by age group and sex, from 1999 to 2014, were obtained from the Division of Cancer Registration & Surveillance in the Korean National Cancer Control Institute [39].

The age-standardized incidence rate (SIR) was calculated using the mid-year Korean registration population in 2000 as the standard population. This method uses the same formula for the regional cancer incidence rate as that used in the Korean Statistical Information Service (KOSIS) [40]. Data on the regional population in Taean, Korea as a whole, and the coastal areas were obtained from the KOSIS during the study period [40].

### 2.3. Statistical Analysis

To compare the SIRs between Taean or the HSOS-exposure areas in Taean and Korea as a whole or the coastal areas, the five- or six-year incidence rates and incidence rate ratios (IRRs) were calculated for 1999−2003, 2004−2008, and 2009−2014. To obtain the 95% confidence interval (CI), the standard error (SE) for the five-year or six-year incidence rate was calculated using a formula proposed by Boyle and Parkin [41]. Additionally, to compare the SIR trends among the areas, the SIRs were calculated per year. For the analyses, the SIR and SE were replaced by 0.0001 when the SIR was 0. The SE for the trend analyses was calculated using the joinpoint regression model formula [42].

To examine the cancer incidence trend in the areas, joinpoint regression analysis was performed. A model selection and average annual percent change (AAPC) comparison test were performed using the Monte Carlo permutation method [42] through the Joinpoint Regression Program version 4.5.0.1 (Bethesda, MD, USA) [43]. Average annual percent change (APC) during the period means that the increase in the cancer incidence rate is equivalent during the period. In the final model, each joinpoint indicates a significant change in the slope and a zero joinpoint implies the absence of a significant change in the slope, that is, the AAPC is the same as the APC [42]. A permutation test that estimates the optimal number of joinpoints was applied after all the analyses, at a significance level of 0.05 [43].

### 2.4. Ethics Statement

This study was approved by the Institutional Review Board of Dankook University Hospital, Cheonan, Korea (DKUH 2015-12-012; DKUH 2017-03-032). According to the Privacy Protect Act in Korea, informed consent is not required for the use of anonymized national statistics datasets.

## 3. Results

### 3.1. Age-Standardized Incidence Rate and Incidence Rate Ratio 

Table 1 presents the five (or six)-year cancer incidence rate and SIR ratio of five major cancers in Taean and the exposed areas (vs. nationwide and in the coastal areas), from 1999 to 2014. In men, the SIRs of prostate cancer in Taean and the high-exposure areas were significantly higher than those in the coastal areas, from 2009 to 2014 (IRR (95% CI), Taean vs. coastal areas = 1.2 (1.01, 1.53); high-exposure area vs. coastal areas = 1.4 (1.01, 1.92)). The SIRs in Taean and the high-exposure areas were higher than those observed nationwide, but this was not significant. In women, the SIRs for leukemia in Taean and the high-exposure areas were higher than those in the other areas, from 2009 to 2014, but this was not significant.

### 3.2. Trend of Cancer Incidence (Taean vs. Nationwide)

**Figure 2:**
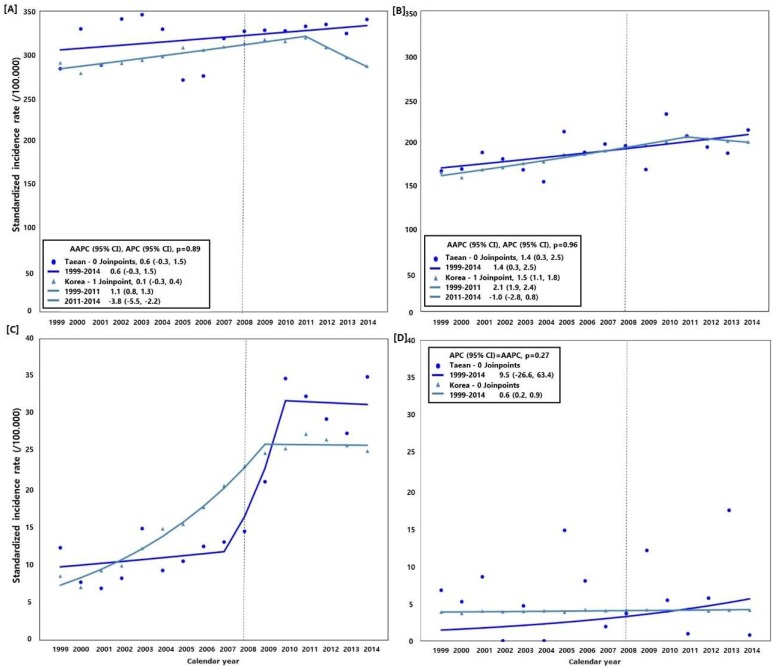
Comparison of the cancer incidence rate and average annual percent change (AAPC) between Taean and the whole of Korea, 1999−2014; Model selection and *p*-value estimated using the Monte Carlo Permutation method. (**A**) All cancers (C00−C96) excluding thyroid cancer (C73) in men; (**B**) All cancers (C00−C96) excluding thyroid cancer (C73) in women; (**C**) Prostate cancer (C61); and (**D**) Leukemia (C91−C95) in women. The Hebei Spirit oil spill (HSOS) occurred in 7 December 2007.

Figure 2 shows the cancer incidence trend in Taean and Korea as a whole from 1999 to 2014. The SIRs of all cancers excluding thyroid cancer showed an increased annual average of 0.6% (95% CI: −0.3, 1.5), 0.1% (95% CI: −0.3, 0.4), 1.4% (95% CI: 0.3, 2.5), and 1.5% (95% CI: 1.1, 1.8), in Taean men, men nationwide, Taean women, and women nationwide, respectively. The SIRs of prostate cancer in men increased at an annual average of 8.1% (95% CI: −4.1, 21.8) in Taean and 8.8% (95% CI: 7.5, 10.1) nationwide, while those of leukemia in women increased at an annual average of 9.5% (95% CI: −26.6, 63.4) in Taean and 0.6% (95% CI: 0.2, 0.9) nationwide. In terms of prostate cancer, from 2007 to 2009 the SIRs increased at an annual average of 39.3% in Taean and 13.5% nationwide, but this was not significant. When the AAPCs of Taean and Korea were compared, the trends were not significantly different except in the case of thyroid and prostate cancer, and leukemia in women.

### 3.3. Trend of Cancer Incidence (Taean vs. the Coastal Areas)

**Figure 3:**
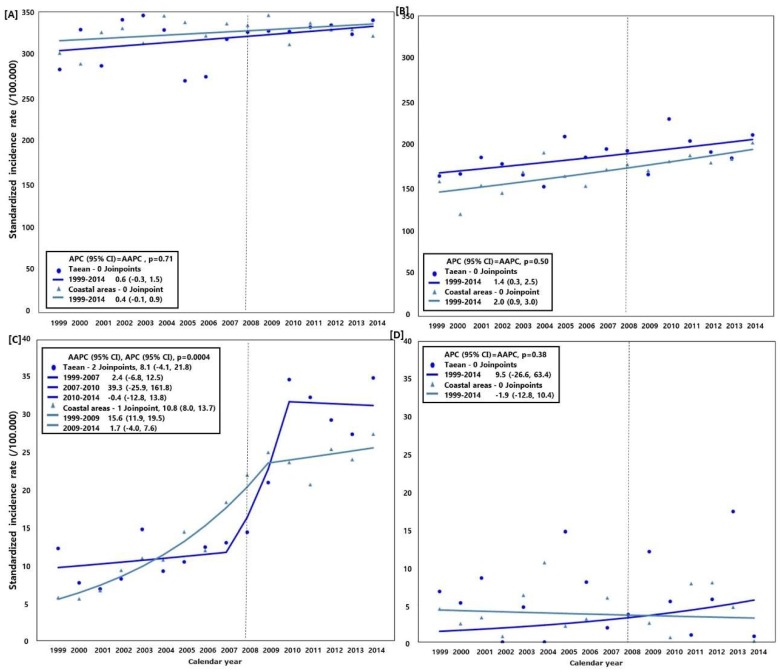
Comparison of the cancer incidence rate and average annual percent change (AAPC) between Taean and the other coastal areas, 1999−2014; Model selection and *p*-value estimated using the Monte Carlo Permutation method. (**A**) All cancers (C00−C96) excluding thyroid cancer (C73) in men; (**B**) All cancers (C00−C96) excluding thyroid cancer (C73) in women; (**C**) Prostate cancer (C61); and (**D**) Leukemia (C91−C95) in women. Coastal areas were selected based on their similarities to Taean in terms of the characteristics and population size. This has been illustrated in Figure 1. The Hebei Spirit oil spill (HSOS) occurred in 7 December 2007.

Figure 3 illustrates the cancer incidence trend in Taean and the coastal areas from 1999 to 2014. The SIRs of all cancers excluding thyroid cancer in the coastal areas increased at an annual average of 0.4% (95% CI: −0.1, 0.9) in men and 2.0% (95% CI: 0.9, 3.0) in women. The SIRs of prostate cancer increased at an annual average of 8.1% (95% CI: −4.1, 21.8) in Taean and 10.8% (95% CI: 8.0, 13.7) in coastal areas, while those of leukemia among women increased at an annual average of 9.5% (95% CI: −26.6, 63.4) in Taean. However, a decrease of 1.9% (95% CI: −12.8, 10.4) was observed in the coastal areas. In terms of prostate cancer, from 2007 to 2009 the SIRs increased at an annual average of 39.3% (95% CI: −25.9, 161.8) in Taean and 15.6% (95% CI: 11.9, 19.5) in the coastal areas. When the AAPCs of Taean and the coastal areas were compared, the trends were significantly different in terms of prostate cancer (*p* = 0.0004) but not in all cancers, excluding thyroid cancer and leukemia among women.

## 4. Discussion

In this study, we found that the incidence rate of prostate cancer has increased in Taean since 2007 and is higher than that observed in other coastal areas, in 2009−2014. Furthermore, the incidence rates were higher in the high-exposure areas than in the low-exposure areas. Additionally, the APC of the incidence rates in Taean was higher than that observed nationwide and in the coastal areas (Taean [2007–2010]: 39.3%; nationwide [1999–2009]: 13.5%; coastal areas [1999–2009]: 15.6%). However, as cancer can develop as late as 10 years after exposure, it may be difficult to conclude that the cancer developed because of exposure to the oil spill. 

In Taean, a coal-fired power plant has been in existence since 1995 [44]; it is located on the same coast 15 km away from the spot of the oil spill [35]. The high-exposure HSOS areas largely overlap with the areas with high exposure to compounds from the power plant. Therefore, it could be assumed that residents previously exposed to the coal power plant may have been exposed to the oil spill too; the interaction between these environmental exposures should be taken into account.

In Korea, the prostate cancer incidence rate has increased since 1999 when the cancer database was set up [45]. According to the Korean National Cancer Control Institute in the National Cancer Center, the prevalence of prostate cancer has increased and the disease is highly prevalent in areas with a high social economic status (SES); additionally, a high screening rate for prostate cancer leads to detection bias [46]. A previous study reported that men with a low educational level have a low risk for prostate cancer [47]. The incidence rate of prostate cancer was higher among firefighters, as observed in a meta-analysis, due to exposure to VOCs and PAHs [48]. 

In Taean, especially in the high-exposure areas, residents have a low SES [49] and the medical facilities are poor and scarce: the numbers of medical doctors in medical institutes per 1000 of the population were 1.25 in 2007 and 1.5 in 2014, in Taean [40], while the corresponding values nationwide were 1.9 in 2007 and 2.23 in 2014 [50]. Additionally, the Korean National Cancer Screening Program includes only the stomach, liver, colon, breast, and cervix, and the overall rate of participation was 36.3% in 2013 [38]. Therefore, there is no obvious evidence of detection bias for the increasing incidence of prostate cancer.

We found that the incidence of leukemia among female Taean residents had increased and was higher than in the other coastal areas and nationwide, in 2009−2014. The incidence rates were higher in the high-exposure areas than the low-exposure areas. However, the IRR was not significantly high because leukemia is not a common disease and also due to the fact that Taean has a small population. Conversely, in men, the leukemia incidence rate decreased in Taean and was lower in the high-exposure areas than the low-exposure areas in 2009−2014. Additionally, the incidence rate (/100,000) was higher among women (7.3) than men (4.7) in 2009–2014 in Taean, but was higher among men than women in the coastal areas, at 5.8, 4.1, 4.9, and 3.9, among Korean men, Korean women, men in coastal areas, and women in coastal areas, respectively (Supplementary Table S1). In this study, the leukemia incidence rate among women, especially those living in high-exposure areas, increased, while that of men decreased. That could be because the incidence rate of leukemia among women younger than 20 years living in high-exposure areas was higher in 2009−2014 (case = 3) than in 2004−2008 (case = 0); the incidence rate of the disease in men was lower in 2009−2014 (case = 0) than in 2004−2008 (case = 1). A previous study reported that hematologic malignancies are generally more commonly observed in men; while this can be generalized to most other cancers worldwide, infant leukemia could be an exception [51]. Nevertheless, the latency period of leukemia after hazard exposure is less than 5 years [52]. One study reported that the risk of developing leukemia was higher among those living near oil and gas exploitation areas compared to those living far away from such areas [53]. Additionally, organic solvents including benzene are well-known to be risk factors for leukemia [54]. Our study has some limitations associated with its small sample size and low observed incidence rate. Studies with a longer follow-up are needed to examine the leukemia incidence trend, along with additional analyses for all hematopoietic cancers including leukemia, the latency time of which is within 5 years after exposure. 

In this study, we selected comparison areas that are similar to Taean in terms of their characteristics, such as the predominance of the farming, fishing, and tourism industries [37,55], and also the population sizes and proportion of elderly adults (in Figure 1). Cancer incidence rates vary between cities and rural areas [56]. In addition, even in terms of rural areas, the SES level and dietary culture may vary between inland and coastal regions, as well as suburban areas and farming/fishing villages. 

In this study, when we compared the cancer incidence rate, excluding that of thyroid cancer, between Taean and Korea as a whole, we found that the incidence rate tended to increase in Taean during all the periods, but not nationwide, since 2011. We excluded thyroid cancer from the analysis because of the potential detection bias associated with thyroid cancer in Korea. Enhanced accessibility to low-cost diagnostic procedures is reported to have increased the incidence of thyroid cancers with carcinomas smaller than 1 cm in size in Korea [57].

We could not investigate the obvious association between oil exposure and cancer incidence in this study. Although we could not identify the reason for the rapid increase in the incidence rate, we found that the incidence rate of prostate cancer was higher among Taean residents than those living in the other coastal areas; the incidence rates were higher in the high-exposure areas, especially after the HSOS. Additionally, we could not prove the aforementioned association because ours is an ecological study. However, our study participants were all Taean residents, and the reliability and validity of our data are high as we used national data. We did not adjust for confounding factors, such as smoking habit, alcohol consumption, exercise status, SES, health status, and family history, or consider exposure factors, such as the duration of clean-up work and frequency of visits to the oil spill site except for the exposure in the living area. Our study period was not long enough for the cancer incidence after exposure to be analyzed. Therefore, studies with a longer follow-up period are needed. Advanced analyses such as age-period-cohort analyses may provide additional clues pertaining to oil spill exposure and cancer development. Despite these limitations, however, this is the first long-term follow-up study to examine the cancer incidence trend according to oil spill exposure using a national cancer registry of all residents exposed to the oil spill. 

The incidence rate of prostate cancer and burden of the disease have been increasing in Korea [45] and worldwide [58]. This can be investigated through advanced research focusing on the birth cohort effect or role of carcinogens. However, although it is important to clarify the reason for the increase in the cancer incidence rate, it is necessary to conduct screening for early detection and provide treatment for residents.

## 5. Conclusions

After the HSOS, an increased incidence rate of cancer was observed among Taean residents, particularly in terms of prostate cancer. Studies with a longer follow-up are needed to accurately identify the association between cancer incidence and oil spill in residents.

## Figures and Tables

**Table 1:** Age-standardized incidence rate and incidence rate ratio of five major cancers and leukemia according to the Hebei Spirit oil spill exposure areas in Taean, Korea, 1999−2014.

Cancer Type	Taean											
	All				High Exposed Area				Low Exposed Area			
	Case ^1^	SIR ^2^	IRR1 ^3^ (95% CI)	IRR2 ^4^ (95% CI)	Case ^1^	SIR ^2^	IRR1 ^3^ (95% CI)	IRR2 ^4^ (95% CI)	Case ^1^	SIR ^2^	IRR1 ^3^ (95% CI)	IRR2 ^4^ (95% CI)
**Male**												
All cancer (C00−C96)												
1999−2003	753	318.0	1.1 (1.0, 1.2)	1.0 (0.9, 1.1)	284	309.7	1.1 (0.9, 1.2)	1.0 (0.9, 1.1)	420	288.8	1.0 (0.9, 1.1)	0.9 (0.8, 1.0)
2004−2008	872	314.6	1.0 (0.9, 1.1)	0.9 (0.8, 1.0)	323	309.2	1.0 (0.9, 1.1)	0.9 (0.8, 1.0)	547	315.2	1.0 (0.9, 1.1)	0.9 (0.8, 1.0)
2009−2014	1430	350.0	1.1 (1.0, 1.1)	1.0 (0.9, 1.1)	557	370.8	1.1 (1.0, 1.2)	1.1 (1.0, 1.2)	873	340.5	1.0 (1.0, 1.1)	1.0 (0.9, 1.1)
All cancer (C00−C96) excluding thyroid (C73)												
1999−2003	748	315.6	1.1 (1.0, 1.2)	1.0 (0.9, 1.1)	283	309.7	1.1 (0.9, 1.2)	1.0 (0.9, 1.1)	416	288.8	1.0 (0.9, 1.1)	0.9 (0.8, 1.0)
2004−2008	845	302.5	1.0 (0.9, 1.1)	0.9 (0.8, 1.0)	315	309.2	1.0 (0.9, 1.1)	0.9 (0.8, 1.0)	528	315.2	1.0 (0.9, 1.1)	0.9 (0.8, 1.0)
2009−2014	1369	325.3	1.1 (1.0, 1.1)	1.0 (0.9, 1.1)	531	370.8	1.1 (1.0, 1.2)	1.0 (0.9, 1.1)	838	340.5	1.0 (1.0, 1.1)	1.0 (0.9, 1.1)
Stomach (C16)												
1999−2003	207	89.6	1.3 (1.1, 1.6)	1.2 (1.0, 1.4)	69	81.4	1.2 (0.9, 1.6)	1.1 (0.8, 1.4)	123	85.2	1.3 (1.0, 1.5)	1.1 (0.9, 1.4)
2004−2008	183	63.9	1.0 (0.8, 1.1)	0.9 (0.7, 1.0)	69	60.2	0.9 (0.7, 1.2)	0.8 (0.6, 1.1)	114	64.1	1.0 (0.8, 1.2)	0.9 (0.7, 1.1)
2009−2014	277	67.1	1.1 (1.0, 1.3)	1.1 (0.9, 1.2)	101	64.4	1.1 (0.8, 1.3)	1.0 (0.8, 1.3)	176	67.5	1.1 (0.9, 1.3)	1.1 (0.9, 1.3)
Lung (C33−C34)												
1999−2003	128	51.2	1.0 (0.8, 1.2)	0.9 (0.7, 1.0)	58	55.1	1.1 (0.8, 1.4)	0.9 (0.7, 1.2)	60	40.3	0.8 (0.6, 1.0)	0.7 (0.5, 0.9)
2004−2008	173	58.0	1.2 (1.0, 1.4)	1.0 (0.8, 1.2)	58	53.3	1.1 (0.8, 1.5)	0.9 (0.7, 1.2)	115	62.8	1.3 (1.0, 1.6)	1.1 (0.9, 1.3)
2009−2014	257	53.2	1.2 (1.0, 1.3)	1.0 (0.9, 1.2)	102	56.3	1.2 (1.0, 1.6)	1.1 (0.9, 1.4)	155	53.1	1.2 (1.0, 1.4)	1.0 (0.9, 1.2)
Colon (C18−C20)												
1999−2003	55	22.7	0.7 (0.6, 0.9)	0.9 (0.6, 1.1)	22	21.3	0.7 (0.5, 1.0)	0.8 (0.5, 1.2)	32	21.7	0.7 (0.5, 0.9)	0.8 (0.6, 1.2)
2004−2008	102	37.8	0.9 (0.7, 1.0)	1.1 (0.8, 1.3)	37	34.8	0.8 (0.6, 1.1)	1.0 (0.7, 1.4)	65	38.2	0.9 (0.7, 1.1)	1.1 (0.8, 1.4)
2009−2014	197	48.7	1.0 (0.9, 1.2)	1.0 (0.9, 1.3)	69	50.2	1.0 (0.8, 1.4)	1.1 (0.8, 1.5)	128	49.0	1.0 (0.8, 1.2)	1.1 (0.9, 1.3)
Liver (C22)												
1999−2003	109	47.8	1.0 (0.9, 1.3)	0.9 (0.7, 1.1)	37	42.3	0.9 (0.7, 1.3)	0.8 (0.6, 1.1)	57	39.9	0.9 (0.7, 1.1)	0.8 (0.6, 1.0)
2004−2008	114	43.6	1.0 (0.9, 1.3)	0.8 (0.7, 1.0)	40	43.0	1.0 (0.7, 1.5)	0.8 (0.6, 1.1)	74	43.8	1.0 (0.8, 1.3)	0.8 (0.7, 1.1)
2009−2014	147	36.9	1.0 (0.9, 1.2)	0.8 (0.7, 1.0)	58	41.2	1.2 (0.8, 1.6)	0.9 (0.7, 1.2)	89	34.9	1.0 (0.8, 1.2)	0.8 (0.6, 0.9)
Prostate (C61)												
1999−2003	27	10.2	1.1 (0.7, 1.6)	1.3 (0.8, 2.1)	15	13.8	1.5 (0.8, 2.7)	1.8 (0.9, 3.6)	12	7.9	0.8 (0.5, 1.4)	1.0 (0.5, 1.9)
2004−2008	36	12.2	0.7 (0.5, 0.9)	0.8 (0.6, 1.1)	14	12.3	0.7 (0.4, 1.0)	0.8 (0.5, 1.3)	22	11.8	0.6 (0.5, 0.9)	0.8 (0.5, 1.1)
2009−2014	143	30.3	1.2 (1.0, 1.4)	1.2 (1.0, 1.5)	62	33.9	1.3 (1.0, 1.8)	1.4 (1.0, 1.9)	81	28.3	1.1 (0.9, 1.4)	1.2 (0.9, 1.5)
Leukemia (C91−C95)												
1999−2003	14	6.6	1.3 (0.7, 2.3)	1.1 (0.6, 2.2)	5	6.6	1.3 (0.4, 3.8)	1.1 (0.4, 3.5)	9	6.6	1.2 (0.6, 2.6)	1.1 (0.5, 2.5)
2004−2008	16	8.9	1.6 (0.8, 3.3)	1.3 (0.6, 2.5)	7	10.6	1.9 (0.5, 7.7)	1.5 (0.4, 5.2)	8	6.8	1.2 (0.6, 2.8)	1.0 (0.4, 2.1)
2009−2014	14	4.7	0.8 (0.4, 1.5)	1.0 (0.5, 2.0)	4	2.2	0.4 (0.2, 0.7)	0.5 (0.2, 1.1)	10	5.6	1.0 (0.5, 2.0)	1.1 (0.5, 2.7)
**Female**												
All cancer (C00−C96)												
1999−2003	474	192.8	1.0 (0.9, 1.2)	1.1 (1.0, 1.3)	159	169.1	0.9 (0.8, 1.1)	1.0 (0.8, 1.2)	288	184.8	1.0 (0.9, 1.2)	1.1 (1.0, 1.3)
2004−2008	679	264.4	1.1 (1.0, 1.2)	1.1 (1.0, 1.3)	233	251.2	1.1 (0.9, 1.3)	1.1 (0.9, 1.3)	446	266.9	1.1 (1.0, 1.2)	1.2 (1.0, 1.3)
2009−2014	905	325.6	1.0 (1.0, 1.1)	1.1 (1.0, 1.2)	358	295.6	1.0 (0.8, 1.1)	1.0 (0.8, 1.2)	738	327.0	1.1 (1.0, 1.2)	1.1 (1.0, 1.2)
All cancer (C00−C96) excluding thyroid (C73)												
1999−2003	434	170.0	1.0 (0.9, 1.2)	1.2 (1.0, 1.3)	148	155.7	0.9 (0.8, 1.2)	1.1 (0.9, 1.3)	259	162.0	1.0 (0.9, 1.1)	1.1 (1.0, 1.3)
2004−2008	527	185.4	1.0 (0.9, 1.1)	1.1 (1.0, 1.2)	188	184.6	1.0 (0.8, 1.2)	1.1 (0.9, 1.3)	338	186.7	1.0 (0.9, 1.1)	1.1 (0.9, 1.3)
2009−2014	805	198.3	1.0 (0.9, 1.1)	1.1 (1.0, 1.2)	270	188.2	0.9 (0.8, 1.2)	1.0 (0.8, 1.3)	535	204.8	1.0 (0.9, 1.1)	1.1 (1.0, 1.3)
Thyroid (C73)												
1999−2003	40	20.2	1.2 (0.8, 1.7)	1.0 (0.7, 1.5)	11	13.3	0.8 (0.4, 1.4)	0.7 (0.4, 1.2)	29	22.7	1.4 (0.9, 2.1)	1.1 (0.7, 1.8)
2004−2008	153	76.7	1.4 (1.1, 1.7)	1.3 (1.1, 1.7)	45	66.6	1.2 (0.8, 1.8)	1.2 (0.8, 1.8)	108	80.2	1.5 (1.1, 1.9)	1.4 (1.1, 1.8)
2009−2014	291	119.3	1.1 (1.0, 1.3)	1.1 (0.9, 1.2)	88	107.4	1.0 (0.8, 1.4)	1.0 (0.7, 1.3)	203	122.2	1.2 (1.0, 1.4)	1.1 (0.9, 1.3)
Breast (C50)												
1999−2003	57	31.9	1.1 (0.8, 1.5)	1.8 (1.2, 2.6)	17	28.1	1.0 (0.6, 1.7)	1.5 (0.8, 3.1)	40	33.4	1.2 (0.8, 1.7)	1.8 (1.2, 2.9)
2004−2008	75	39.3	1.0 (0.8, 1.3)	1.4 (1.0, 1.9)	25	42.1	1.1 (0.6, 1.9)	1.4 (0.8, 2.8)	50	38.7	1.0 (0.7, 1.3)	1.3 (0.9, 1.9)
2009−2014	118	46.4	0.9 (0.8, 1.1)	1.4 (1.0, 1.8)	35	39.5	0.8 (0.5, 1.2)	1.2 (0.7, 1.9)	83	47.6	1.0 (0.8, 1.2)	1.4 (1.0, 1.9)
Colon (C18−C20)												
1999−2003	60	20.9	1.1 (0.8, 1.5)	1.3 (0.9, 1.9)	16	11.8	0.6 (0.4, 1.0)	0.7 (0.4, 1.2)	42	24.5	1.3 (0.9, 1.9)	1.5 (1.0, 2.3)
2004−2008	66	21.0	0.9 (0.7, 1.1)	0.9 (0.6, 1.2)	22	19.9	0.8 (0.5, 1.4)	0.8 (0.5, 1.4)	44	21.8	0.9 (0.7, 1.2)	0.9 (0.6, 1.3)
2009−2014	130	23.0	0.9 (0.7, 1.0)	0.9 (0.7, 1.2)	39	17.9	0.7 (0.5, 0.9)	0.7 (0.5, 1.0)	91	25.7	1.0 (0.8, 1.2)	1.0 (0.8, 1.4)
Stomach (C16)												
1999−2003	82	29.1	1.1 (0.8, 1.4)	1.1 (0.8, 1.4)	28	29.4	1.1 (0.6, 1.8)	1.1 (0.6, 1.9)	49	27.3	1.0 (0.7, 1.3)	1.0 (0.7, 1.4)
2004−2008	96	31.3	1.2 (0.9, 1.5)	1.1 (0.8, 1.5)	32	25.3	0.9 (0.6, 1.6)	0.9 (0.5, 1.5)	64	34.0	1.3 (0.9, 1.7)	1.2 (0.9, 1.7)
2009−2014	139	31.6	1.2 (1.0, 1.6)	1.1 (0.9, 1.5)	46	26.8	1.1 (0.6, 1.7)	1.0 (0.6, 1.6)	93	33.4	1.3 (1.0, 1.8)	1.2 (0.9, 1.6)
Lung (C33−C34)												
1999−2003	46	14.5	1.2 (0.8, 1.6)	1.1 (0.8, 1.7)	19	11.8	0.9 (0.6, 1.5)	0.9 (0.6, 1.5)	22	12.7	1.0 (0.6, 1.6)	1.0 (0.6, 1.6)
2004−2008	60	15.4	1.1 (0.8, 1.5)	1.4 (1.0, 2.1)	18	11.9	0.9 (0.5, 1.4)	1.1 (0.6, 2.0)	42	18.0	1.3 (0.9, 1.9)	1.7 (1.1, 2.6)
2009−2014	90	15.3	1.0 (0.8, 1.3)	1.2 (0.8, 1.6)	37	19.8	1.3 (0.7, 2.6)	1.5 (0.7, 3.1)	53	14.3	1.0 (0.7, 1.3)	1.1 (0.8, 1.5)
Leukemia (C91−C95)												
1999−2003	11	5.1	1.3 (0.6, 2.8)	1.5 (0.6, 3.7)	2	1.4	0.4 (0.2, 0.8)	0.4 (0.1, 1.2)	7	5.0	1.3 (0.5, 3.2)	1.5 (0.5, 4.2)
2004−2008	13	5.7	1.4 (0.6, 3.1)	1.1 (0.5, 2.5)	6	10.8	2.7 (0.5, 15.2)	2.1 (0.4, 10.3)	7	3.9	1.0 (0.4, 2.2)	0.8 (0.3, 1.9)
2009−2014	13	7.3	1.8 (0.7, 4.5)	1.9 (0.7, 5.0)	6	20.8	5.1 (0.7, 36.3)	5.3 (0.7, 39.1)	7	4.2	1.0 (0.4, 2.8)	1.1 (0.3, 3.3)

^1^ The number of cases in Taean may vary in terms of the sum of the high-exposure and low-exposure areas, in situations in which address details were absent; ^2^ Standardized incidence rate: Rate per 100,000; The standard population was the Korean mid-year population in 2000; ^3^ The standardized incidence rate ratio was calculated as the standardized incidence rate in Taean divided by that in Korea and the 95% CI was estimated using the standardized incidence rate and the formula proposed by Boyle P. and Parkin D.M (“Statistical methods for registries” from book of “Cancer Registration: Principles and Methods” (IARC Scientific Publications No. 95)); ^4^ The standardized incidence rate ratio was calculated as the standardized incidence rate in Taean divided by that in the coastal area and the 95% CI was estimated using the standardized incidence rate and the above-stated formula. The coastal areas were selected based on their similarities to Taean, in terms of their characteristics and population size. This has been illustrated in Figure 1. CI, confidence interval; SIR, standardized incidence rate; IRR, incidence rate ratio.

## Supplementary Materials

The following are available online at http://www.mdpi.com/1660-4601/15/5/1006/s1, Table S1: Age-standardized incidence rate of five major cancer in Korea and coastal areas, 1999-2014.

## References

[1] Fingas M. (2017). Oil Spill Science and Technology.

[2] Choi K.H., Lim M.H., Ha M., Sohn J.N., Kang J.W., Choi Y.H., Cheong H.K. (2015). Psychological vulnerability of residents of communities affected by the Hebei spirit oil spill. Disaster Med. Public Health Prep..

[3] Cheong H.K., Ha M., Lee J.S., Kwon H., Ha E.H., Hong Y.C., Choi Y., Jeong W.C., Hur J., Lee S.M. (2011). Hebei spirit oil spill exposure and subjective symptoms in residents participating in clean-up activities. Environ. Health Toxicol..

[4] International Agency for Research on Cancer (IARC) (2018). IARC Monographs on the Evaluation of Carcinogenic Risks to Humans.

[5] Ha M., Kwon H., Cheong H.K., Lim S., Yoo S.J., Kim E.J., Park S.G., Lee J., Chung B.C. (2012). Urinary metabolites before and after cleanup and subjective symptoms in volunteer participants in cleanup of the Hebei spirit oil spill. Sci. Total Environ..

[6] Kim B.-M., Park E.-K., LeeAn S.-Y., Ha M.-N., Kim E.-J., Kwon H.-J., Hong Y.-C., Jeong W.-C., Hur J.-I., Cheong H.-K. (2009). Btex exposure and its health effects in pregnant women following the Hebei spirit oil spill. J. Prev. Med. Public Health.

[7] Lee C.-H., Kang Y.-A., Chang K.-J., Kim C.-H., Hur J.-I., Kim J.-Y., Lee J.-K. (2010). Acute health effects of the Hebei spirit oil spill on the residents of taean, Korea. J. Prev. Med. Public Health.

[8] Lee S.-M., Ha M.-N., Kim E.-J., Jeong W.-C., Hur J.-I., Park S.-G., Kwon H.-J., Hong Y.-C., Ha E.-H., Lee J.-S. (2009). The effects of wearing protective devices among residents and volunteers participating in the cleanup of the Hebei spirit oil spill. J. Prev. Med. Public Health.

[9] Lee I.-J., Jang B.-K., Lee J.-W., Son B.-S., Cheong H.-K., Ha M., Choi Y.-H., Park M. (2015). Association between metabolic syndrome and participation in clean-up work at the Hebei spirit oil spill. Korean J. Environ. Health Sci..

[10] Song M.-K., Hong Y.-C., Cheong H.-K., Ha M.-N., Kwon H.-J., Ha E.-H., Choi Y., Jeong W.-C., Hur J., Lee S.-M. (2009). Psychological health in residents participating in clean-up works of Hebei spirit oil spill. J. Prev. Med. Public Health.

[11] Ha M., Jeong W.-C., Lim M., Kwon H., Choi Y., Yoo S.-J., Noh S.R., Cheong H.-K. (2013). Children’s mental health in the area affected by the Hebei spirit oil spill accident. Environ. Health Toxicol..

[12] Eum J.-H., Cheong H.-K., Ha M., Kwon H.-J., Ha E.-H., Hong Y.-C., Choi Y.Y., Jeong W.C., Hur J.-I., Lee S. (2008). Hebei spirit oil spill exposure and acute neuropsychiatric effects on residents participating in clean-up work. Epidemiol. Health.

[13] Noh S.R., Cheong H.-K., Ha M., Eom S.-Y., Kim H., Choi Y.-H., Paek D. (2015). Oxidative stress biomarkers in long-term participants in clean-up work after the Hebei spirit oil spill. Sci. Total Environ..

[14] Kim J.A., Noh S.R., Cheong H.K., Ha M., Eom S.Y., Kim H., Park M.S., Chu Y., Lee S.H., Choi K. (2017). Urinary oxidative stress biomarkers among local residents measured 6 years after the Hebei spirit oil spill. Sci. Total Environ..

[15] Hildur K., Templado C., Zock J.P., Giraldo J., Pozo-Rodriguez F., Frances A., Monyarch G., Rodriguez-Trigo G., Rodriguez-Rodriguez E., Souto A. (2015). Follow-up genotoxic study: Chromosome damage two and six years after exposure to the prestige oil spill. PLoS ONE.

[16] Frances A., Hildur K., Barbera J.A., Rodriguez-Trigo G., Zock J.P., Giraldo J., Monyarch G., Rodriguez-Rodriguez E., de Castro Reis F., Souto A. (2016). Persistence of breakage in specific chromosome bands 6 years after acute exposure to oil. PLoS ONE.

[17] Rodriguez-Trigo G., Zock J.P., Pozo-Rodriguez F., Gomez F.P., Monyarch G., Bouso L., Coll M.D., Verea H., Anto J.M., Fuster C. (2010). Health changes in fishermen 2 years after clean-up of the prestige oil spill. Ann. Intern. Med..

[18] Wickliffe J.K., Simon-Friedt B., Howard J.L., Frahm E., Meyer B., Wilson M.J., Pangeni D., Overton E.B. (2018). Consumption of fish and shrimp from southeast louisiana poses no unacceptable lifetime cancer risks attributable to high-priority polycyclic aromatic hydrocarbons. Risk Anal..

[19] Palinkas L.A., Petterson J.S., Russell J.C., Downs M.A. (2004). Ethnic differences in symptoms of post-traumatic stress after the exxon valdez oil spill. Prehosp. Disaster Med..

[20] Arata C.M., Picou J.S., Johnson G.D., McNally T.S. (2000). Coping with technological disaster: An application of the conservation of resources model to the exxon valdez oil spill. J. Trauma. Stress.

[21] Palinkas L.A., Russell J., Downs M.A., Petterson J.S. (1992). Ethnic differences in stress, coping, and depressive symptoms after the exxon valdez oil spill. J. Nerv. Ment. Dis..

[22] Buttke D., Vagi S., Bayleyegn T., Sircar K., Strine T., Morrison M., Allen M., Wolkin A. (2012). Mental health needs assessment after the gulf coast oil spill-Alabama and Mississippi, 2010. Prehosp. Disaster Med..

[23] Buttke D., Vagi S., Schnall A., Bayleyegn T., Morrison M., Allen M., Wolkin A. (2012). Community assessment for public health emergency response (casper) one year following the gulf coast oil spill: Alabama and Mississippi, 2011. Prehosp. Disaster Med..

[24] Buckingham-Howes S., Holmes K., Glenn Morris J., Grattan L.M. (2018). Prolonged financial distress after the deepwater horizon oil spill predicts behavioral health. J. Behav. Health Serv. Res..

[25] Rung A.L., Oral E., Fontham E., Harrington D.J., Trapido E.J., Peters E.S. (2018). The long-term effects of the deepwater horizon oil spill on women’s depression and mental distress. Disaster Med. Public Health Prep..

[26] Kwok R.K., McGrath J.A., Lowe S.R., Engel L.S., Jackson W.B.N., Curry M.D., Payne J., Galea S., Sandler D.P. (2017). Mental health indicators associated with oil spill response and clean-up: Cross-sectional analysis of the gulf study cohort. Lancet Public Health.

[27] Cherry K.E., Sampson L., Galea S., Marks L.D., Baudoin K.H., Nezat P.F., Stanko K.E. (2017). Health-related quality of life in older coastal residents after multiple disasters. Disaster Med. Public Health Prep..

[28] Rung A.L., Gaston S., Oral E., Robinson W.T., Fontham E., Harrington D.J., Trapido E., Peters E.S. (2016). Depression, mental distress, and domestic conflict among louisiana women exposed to the deepwater horizon oil spill in the watch study. Environ. Health Perspect..

[29] Schulenberg S.E., Smith C.V., Drescher C.F., Buchanan E.M. (2016). Assessment of meaning in adolescents receiving clinical services in Mississippi following the deepwater horizon oil spill: An application of the purpose in life test-short form (PIL-SF). J. Clin. Psychol..

[30] Gam K.B., Kwok R.K., Engel L.S., Curry M.D., Stewart P.A., Stenzel M.R., McGrath J.A., Jackson W.B., Jensen R.L., Keil A.P. (2018). Lung function in oil spill response workers 1–3 years after the deepwater horizon disaster. Epidemiology.

[31] Gam K.B., Kwok R.K., Engel L.S., Curry M.D., Stewart P.A., Stenzel M.R., McGrath J.A., Jackson W.B., Jensen R.L., Lichtveld M.Y. (2018). Exposure to oil spill chemicals and lung function in deepwater horizon disaster response workers. J. Occup. Environ. Med..

[32] Alexander M., Engel L.S., Olaiya N., Wang L., Barrett J., Weems L., Schwartz E.G., Rusiecki J.A. (2018). The deepwater horizon oil spill coast guard cohort study: A cross-sectional study of acute respiratory health symptoms. Environ. Res..

[33] McGowan C.J., Kwok R.K., Engel L.S., Stenzel M.R., Stewart P.A., Sandler D.P. (2017). Respiratory, dermal, and eye irritation symptoms associated with corexit EC9527A/EC9500A following the deepwater horizon oil spill: Findings from the gulf study. Environ. Health Perspect..

[34] Kim Y.M., Park J.H., Choi K., Noh S.R., Choi Y.H., Cheong H.K. (2013). Burden of disease attributable to the Hebei spirit oil spill in taean, Korea. BMJ Open.

[35] Google Maps (2018). Google Maps. https://www.google.com/maps.

[36] Kahle D., Wickham H. (2013). Ggmap: Spatial visualization with ggplot2. R J..

[37] Kim S.-Y., Jeong D.-M., Kim K.-M. (2008). Classification Method Reflecting Various Characteristics of Rural Areas.

[38] Korean National Cancer Center (2015). Cancer Facts and Figures 2015.

[39] Korean National Cancer Center Organization of National Cancer Control Institute. http://www.ncc.re.kr/main.ncc?uri=english/sub04_Organization.

[40] Korean Statistical Information Service (KOSIS) Statistical Datatbase.

[41] Boyle P., Parkin D.M. (1991). Statistical methods for registries. Cancer Registration: Principles and Methods.

[42] Kim H.J., Fay M.P., Feuer E.J., Midthune D.N. (2000). Permutation tests for joinpoint regression with applications to cancer rates. Stat. Med..

[43] Statistical Research and Application Branch, National Cancer Institute (2017). Joinpoint Regression Program, Version 4.5.0.1.

[44] Korean Western Power Co., L. History of Taean Power Plant. https://www.iwest.co.kr/bsn/sub.asp?mid=1326.

[45] Jung K.-W., Won Y.-J., Kong H.-J., Lee E.S. (2018). Cancer statistics in Korea: Incidence, mortality, survival, and prevalence in 2015. Cancer Res. Treat..

[46] Korea Central Cancer Registry, N.C.C., National Health and Welfare (2016). The First Announcement of Cancer occurrence Statistics and Occurrence Map by City and District in Korea.

[47] Merletti F., Galassi C., Spadea T. (2011). The socioeconomic determinants of cancer. Environ. Health Glob. Access Sci. Source.

[48] Sritharan J., Pahwa M., Demers P.A., Harris S.A., Cole D.C., Parent M.E. (2017). Prostate cancer in firefighting and police work: A systematic review and meta-analysis of epidemiologic studies. Environ. Health Glob. Access Sci. Source.

[49] Jeon Y.-J., Jang B.-K., Lee I.-J., Lee J.-W., Son B.-S., Cheong H.-K., Ha M., Choi Y.-H., Park M., Lee S.-H. (2016). Impact of allergic diseases in elementary school students by the Hebei spirit oil spill. Korean Public Health Res..

[50] Ministry of Health and Welfare (2017). The Trend of Medical Staff and Facillity in 2007–2016.

[51] Dorak M.T., Karpuzoglu E. (2012). Gender differences in cancer susceptibility: An inadequately addressed issue. Front. Genet..

[52] Nadler D.L., Zurbenko I.G. (2014). Estimating cancer latency times using a weibull model. Adv. Epidemiol..

[53] McKenzie L.M., Allshouse W.B., Byers T.E., Bedrick E.J., Serdar B., Adgate J.L. (2017). Childhood hematologic cancer and residential proximity to oil and gas development. PLoS ONE.

[54] Polychronakis I., Dounias G., Makropoulos V., Riza E., Linos A. (2013). Work-related leukemia: A systematic review. J. Occup. Med. Toxicol..

[55] Kim Y.M., Cheong H.-K., Kim J.H., Kim J.H., Ko K., Ha M. (2009). Scientific basis of environmental health contingency planning for a coastal oil spill. J. Prev. Med. Public Health.

[56] Song H.N., Go S.I., Lee W.S., Kim Y., Choi H.J., Lee U.S., Kang M.H., Lee G.W., Kim H.G., Kang J.H. (2016). Population-based regional cancer incidence in Korea: Comparison between urban and rural areas. Cancer Res. Treat..

[57] Chung J.H. (2014). Unfounded reports on thyroid cancer. J. Korean Med. Sci..

[58] Pishgar F., Ebrahimi H., Saeedi Moghaddam S., Fitzmaurice C., Amini E. (2018). Global, regional and national burden of prostate cancer, 1990 to 2015: Results from the global burden of disease study 2015. J. Urol..

