# Erratum: Choi, K.-H., et al. Cancer Incidence Trend in the Hebei Spirit Oil Spill Area, from 1999 to 2014: An Ecological Study. *Int. J. Environ. Res. Public Health* 2018, *15*, 1006

**DOI:** 10.3390/ijerph15071448

**Published:** 2018-07-09

**Authors:** Kyung-Hwa Choi, Myung-Sook Park, Mina Ha, Jong-Il Hur, Hae-Kwan Cheong

**Affiliations:** 1 Taean Environmental Health Center, Taean, Chungnam 32148, Korea; rosach72@hanmail.net (K.-H.C.); pms1816@gmail.com (M.-S.P.); gsjongil@korea.kr (J.-I.H.); 2 Department of Preventive Medicine, Dankook University College of Medicine, Cheonan, Chungnam 31116, Korea; minaha@dku.edu; 3 Department of Social and Preventive Medicine, Sungkyunkwan University School of Medicine, Suwon, Gyeonggi 16419, Korea

Due to an error during production, the legend presented in Figure 2 in the Results section of the published paper [1] were incorrect. A corrected version of this figure is provided below. Importantly, these changes do not modify the significance of the results and the related conclusions. The authors would like to apologize for any inconvenience to the readers caused by this error. The article will be updated and the original will remain on the webpage.

## Figures and Tables

**Figure 2:**
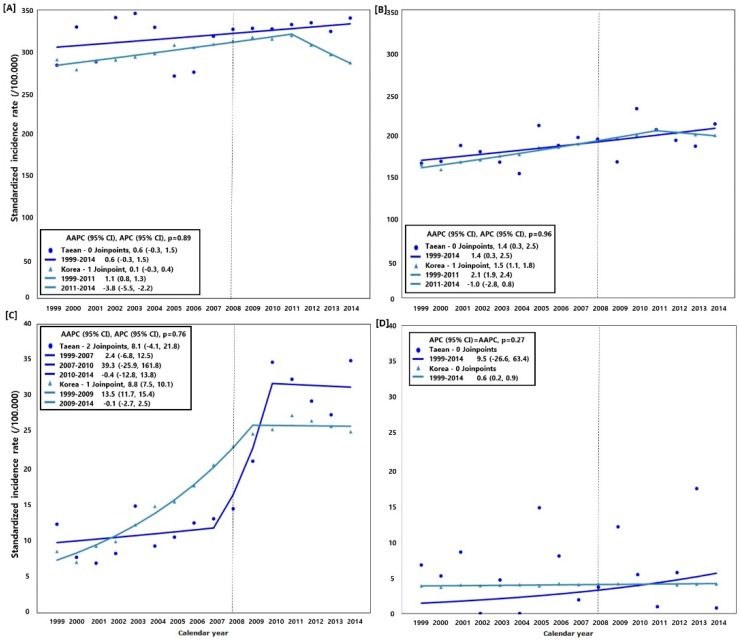
Comparison of the cancer incidence rate and average annual percent change (AAPC) between Taean and the whole of Korea, 1999−2014; Model selection and *p*-value estimated using the Monte Carlo Permutation method. (**A**) All cancers (C00−C96) excluding thyroid cancer (C73) in men; (**B**) All cancers (C00−C96) excluding thyroid cancer (C73) in women; (**C**) Prostate cancer (C61) in men; and (**D**) Leukemia (C91−C95) in women. The Hebei Spirit oil spill (HSOS) occurred in 7 December 2007.

## References

[1] Choi K.H., Park M.S., Ha M., Hur J.I., Cheong H.K. (2018). Cancer incidence trend in the hebei spirit oil spill area, from 1999 to 2014: An ecological study. Int. J. Environ. Res. Public Health.

