# Supplementary material for: Cancer Incidence Trend in the Hebei Spirit Oil Spill Area, from 1999 to 2014: An Ecological Study
Source: Int J Environ Res Public Health. 2018 May 17;15(5):1006. doi: 10.3390/ijerph15051006 (PMC5982045; doi:10.3390/ijerph15051006)
Supplement: Supplementary file 1 [file ijerph-15-01006-s001.pdf]

Table S1. Age-standardized incidence rate of five major cancer in Korea and coastal areas, 1999-2014.

	**Korea nationwide**		**Coastal areas**	
Cancer type	Case	Standardized incidence rate	Case	Standardized incidence rate
Male				
All cancer (C00-C96)				
1999-2003	313,555	291.0	3,178	314.3
2004-2008	419,518	317.3	3,629	344.5
2009-2014	659,644	330.5	4,892	350.2
All cancer (C00-C96) excluding thyroid (C73)				
1999-2003	310,173	288.1	3,155	311.2
2004-2008	406,467	307.5	3,570	335.8
2009-2014	618,132	306.8	4,737	329.4
Stomach (C16)				
1999-2003	73,343	67.7	770	76.0
2004-2008	87,742	65.7	775	72.6
2009-2014	123,619	60.8	895	63.7
Lung (C33-C34)				
1999-2003	52,196	50.6	649	59.6
2004-2008	63,977	49.2	700	58.2
2009-2014	93,284	45.4	870	50.8
Colon (C18-C20)				
1999-2003	33,479	31.0	269	26.3
2004-2008	58,411	43.8	381	35.5
2009-2014	99,508	49.1	642	46.4
Liver (C22)				
1999-2003	51,452	45.9	486	51.8
2004-2008	57,142	41.9	505	52.7
2009-2014	73,054	35.6	596	45.9
Prostate (C61)				
1999-2003	9,228	9.5	92	7.7
2004-2008	23,825	18.6	208	15.7
2009-2014	53,441	26.0	434	24.3
Leukemia (C91-C95)				
1999-2003	6,041	5.3	44	5.8
2004-2008	6,826	5.5	41	7.1
2009-2014	9,799	5.8	54	4.9
Female				
All cancer (C00-C96)				
1999-2003	243,297	181.2	1,849	166.4
2004-2008	366,747	238.8	2,492	228.9
2009-2014	636,552	304.3	3,599	296.6
All cancer (C00-C96) excluding thyroid (C73)				

1999-2003	222,681	164.5	1,707	146.4
2004-2008	291,858	183.9	2,104	171.6
2009-2014	449,413	198.6	2,828	183.6
Thyroid (C73)				
1999-2003	20,616	16.7	142	20.0
2004-2008	74,889	54.9	388	57.3
2009-2014	187,139	105.7	771	112.9
Breast (C50)				
1999-2003	35,168	28.3	130	18.2
2004-2008	54,987	38.6	194	29.1
2009-2014	96,581	49.9	302	33.7
Colon (C18-C20)				
1999-2003	26,051	18.6	210	16.1
2004-2008	40,800	24.4	328	24.3
2009-2014	65,382	26.4	438	24.6
Stomach (C16)				
1999-2003	38,186	27.5	359	26.7
2004-2008	43,909	26.9	377	28.4
2009-2014	60,266	25.3	496	27.7
Lung (C33-C34)				
1999-2003	18,640	12.6	192	12.7
2004-2008	24,952	13.9	201	10.7
2009-2014	40,039	15.0	297	13.3
Leukemia (C91-C95)				
1999-2003	4,819	3.8	26	3.3
2004-2008	5,394	4.0	35	5.1
2009-2014	7,522	4.1	43	3.9
